# Immunohistochemical study of annular erythema appearing in a patient with sub‐acute cutaneous lupus erythematosus

**DOI:** 10.1002/ski2.124

**Published:** 2022-06-15

**Authors:** Kaori Shima, Takashi Nomura, Satoru Yonekura, Yuki Honda Keith, Toshiaki Kogame, Kosaku Murakami, Kenji Kabashima

**Affiliations:** ^1^ Department of Dermatology Kyoto University Graduate School of Medicine Kyoto Japan; ^2^ Center for Cancer Immunotherapy and Immunobiology Kyoto University Graduate School of Medicine Kyoto Japan; ^3^ Singapore Immunology Network (SIgN) Skin Research Institute of Singapore (SRIS) Agency for Science, Technology and Research (A*STAR) Singapore Singapore

## Abstract

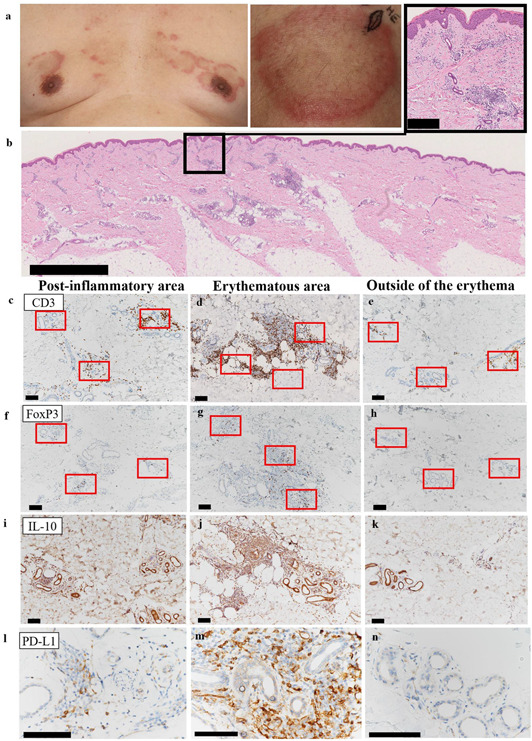

Dear Editor,

1

Annular erythema (AE) is one of the clinical manifestations as annular variant of sub‐acute cutaneous lupus erythematosus (SCLE). To our best knowledge, there have been no reports explaining the formation of an annular pattern with central healing from the eruption. Therefore, we analysed the pattern of infiltrating immune cells in an AE lesion in a patient with SCLE.

A 60‐year‐old man with a history of hypertension was referred to our clinic for multiple AEs that had developed for 3 months. He was consuming irbesartan, amlodipine besylate, and rabeprazole. Blood tests showed elevated aspartate aminotransferase and alanine aminotransferase levels (61 IU/L [normal range, 12–30] and 128 IU/L [10–42], respectively) and high titres of antinuclear antibody (1:640 [<40]), anti‐SS‐A (>1200 U/mL [<10.0]), and anti‐SS‐B (>1000 U/mL [<10.0]). Test results for anti‐double and single‐stranded DNA antibodies were negative. Physical examination revealed asymptomatic AE with raised dike‐shaped margins on the head, chest, and extremities (Figure 1a). The erythema was not migratory, and there was no evidence of muscle weakness or dryness. A skin biopsy was performed from the AE of the left thigh along the markings (Figure 1a). The histological results showed superficial and deep perivascular and periadnexal inflammation with vacuolar changes. The perivascular and periadnexal infiltration of inflammatory cells was less in the central area of the AE, where the erythema was fading and was considered post‐inflammatory. A few inflammatory cells infiltrated the outside of the erythema. While the vacuolar changes at the interface were consistent with lupus erythematosus (LE), the patient did not meet any of the diagnostic criteria for systemic LE (SLE), Sjogren's syndrome, or dermatomyositis. We diagnosed the patient with SCLE based on the clinical and blood tests and histopathological findings.

**FIGURE 1:**
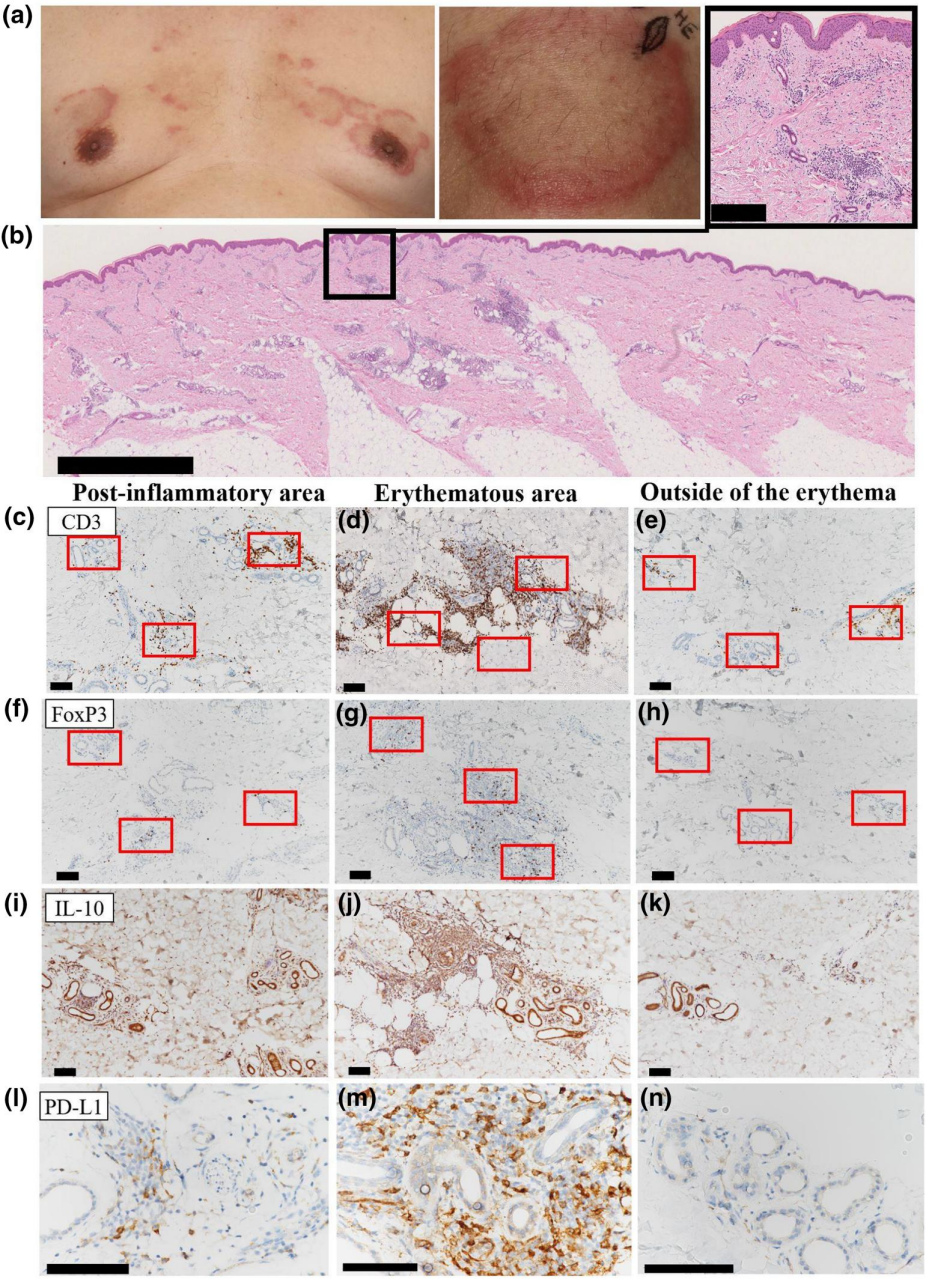
Clinical and histological findings. (a) Clinical appearance of the annular erythematous lesion on the chest and spindle‐shaped marking showing the biopsy site on left thigh. (b) Whole image of the biopsied skin (haematoxylin‐eosin). The scale bar indicates 2.5 mm. The scale bar in the magnified view of the black box indicates 250 µm. (c–e) Immunostaining of CD3, (f–h) FoxP3, (i–k) IL‐10, and (l–n) PD‐L1 of post‐inflammatory site of the inside of erythema, erythematous area, and outside of the erythema. Scale bar = 100 µm

We hypothesised that active immunosuppressive mechanisms became operative in the central post‐inflammatory area by forkhead box P3 (FoxP3^+^) regulatory T cells (Tregs), IL‐10‐producing immunosuppressive cells, or cells participating in the programmed cell death protein 1 (PD‐1)/PD ligand 1 (PD‐L1) axis. Thus, we immunohistochemically investigated the density of infiltrating Tregs (FoxP3^+^ CD3^+^) and the expression of interleukin‐10 (IL‐10) and PD‐L1 in the post‐inflammatory area (Figure 1c,f,i,l), and inside (Figure 1d,g,j,m) and outside (Figure 1e,h,k,n) of the erythematous area. T cells infiltrated the erythematous area most densely (Figure 1d) and in the post‐inflammatory area at a lower density (Figure 1c). Only a few T cells were present outside the erythematous area (Figure 1e). The average numbers of CD3^+^ cells in the three red boxes shown in Figure 1c–e were 83/area (standard deviation = 66.0), 272 (46.2), and 31 (22.7) in the post‐inflammatory area, erythematous area, and outside, respectively. The numbers of FoxP3^+^ were 7.0 (3.0), 46 (24), and 1.3 (1.5), respectively. The average ratio of FoxP3^+^ cells to CD3^+^ cells was 15% (13.0), 17% (7.6), and 9% (14.0), respectively. IL‐10 and PD‐L1 were expressed in the erythematous area at the strongest level (Figure 1j,m) and in the post‐inflammatory area to a lesser extent (Figure 1i,l), but not in the outside (Figure 1k,n). In the post‐inflammatory area, IL‐10 was stained over the entire area in the presence of clearly positive spindle‐shaped cells, suggesting the presence of IL‐10‐producing cells and deposition of IL‐10 in the extracellular matrix. Therefore, inflammation of the central region of AE was considered to be actively suppressed by the mechanism mediated by Tregs, IL‐10^+^ cells, and PD‐L1^+^ cells.

Similar to the other sub‐types of cutaneous lupus erythematosus, photosensitivity and drugs (proton pump inhibitors, antihypertensives, and antifungals) can trigger SCLE.^1^, ^2^ Our case was unlikely to be drug‐induced because the AE persisted after the termination of the medications. Other studies have shown that SCLE patients present a higher percentage of Tregs in the skin than patients with SLE or DLE and healthy donors.^3^ Accordingly, our study revealed an increased number of Tregs in the erythematous and central areas of AE in a patient with SCLE. Such Tregs may be involved in the formation of AE in SCLE compared to other CLE sub‐types.^4^ Apart from FoxP3^+^ regulatory T cells, IL‐10^+^ cells and PD‐L1^+^ cells were yet to be identified in this study due to immunohistochemistry limitations.

AE is a unique clinical finding wherein the centre of the lesion shows spontaneous healing. We assume that multiple regulatory mechanisms are involved in this process. Analysing other forms of AE, including Sjögren's syndrome, centrifugal erythema annulare, and erythema gyratum repens, would help elucidate the immunological mechanisms of these diseases and understand LE pathogenesis.

## AUTHOR CONTRIBUTIONS

**Kaori Shima**: Conceptualization (supporting); Writing – original draft (equal); Writing – review and editing (equal); Investigation – conducting investigative process, performing the experiments, and data/evidence collection (lead); Data curation; Maintain research data (lead); Formal analysis; Application of statistical, mathematical, computational, or other formal techniques to analyze or synthesize study data (supporting). **Takashi Nomura**: Conceptualization (equal); Writing – original draft (equal); Writing – review and editing (equal); Investigation – interpreting the collected data/evidence (lead); Methodology – Development of methodology; Creation of models (supporting). **Satoru Yonekura**: Conceptualization (supporting); Writing – original draft (supporting); Writing – review and editing (supporting); Investigation – interpreting the collected data/evidence (supporting); Methodology – Development of methodology; Creation of models (lead). **Yuki Honda Keith**: Writing – original draft (supporting); Writing – review and editing (supporting). **Toshiaki Kogame**: Writing – original draft (supporting); Writing – review and editing (supporting). **Kosaku Murakami**: Writing – original draft (supporting); Writing – review and editing (supporting). **Kenji Kabashima**: Conceptualization (equal); Writing – original draft (supporting); Writing – review and editing (supporting); Supervision – Oversight and leadership responsibility for the research activity planning and execution, including mentorship external to the core team (lead).

## CONFLICT OF INTEREST

The authors declared that they have no conflicts of interest to this work.

## FUNDING INFORMATION

Health and Labour Sciences Research Grant from the Ministry of Health, Labour and Welfare of Japan, Grant/Award Number: 20FC1035; Kose Cosmetology Research Foundation, Grant/Award Number: AMED‐CREST (JP20gm1210006); Takeda Science Foundation, Grant/Award Numbers: 20H05697, JP20K08649.

## Data Availability

The data that support the findings of this study are available from the corresponding author upon reasonable request after approval from the Ethical Committee of the Kyoto University Hospital.
